# Supplementary material for: The dynamic response of quorum sensing to density is robust to signal supplementation and individual signal synthase knockouts
Source: Microbiology (Reading). 2023 May 19;169(5):001321. doi: 10.1099/mic.0.001321 (PMC10268839; doi:10.1099/mic.0.001321)
Supplement: Supplementary material 1 [file mic-169-1321-s001.pdf]

Supplemental Materials

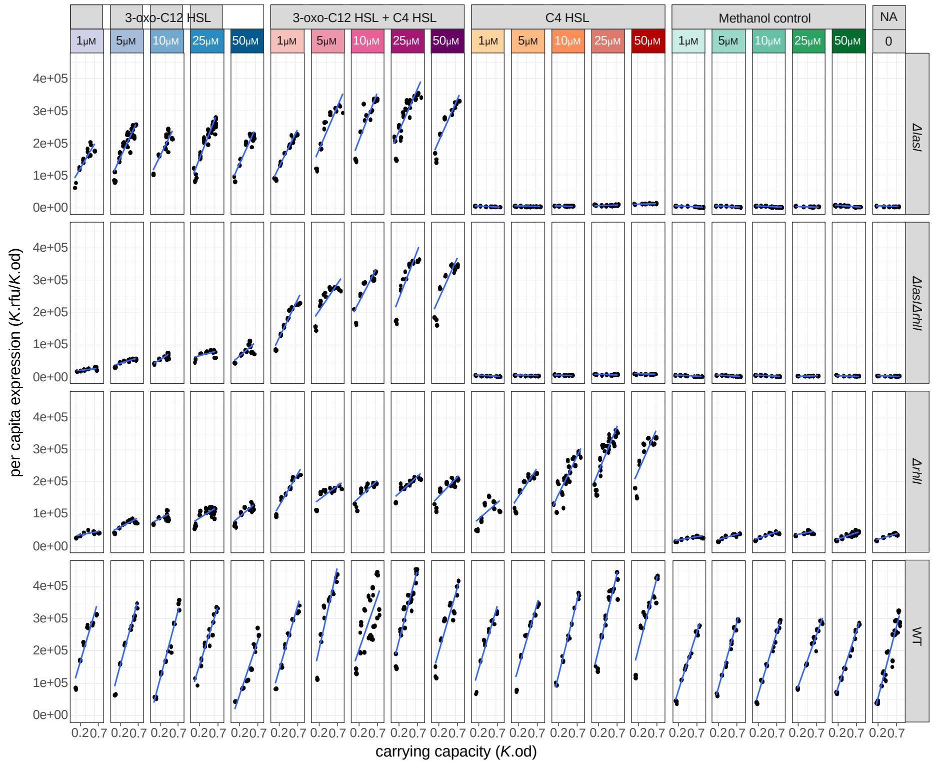

Supplemental Figure 1. Data and linear model fits. Data (black circles) with linear model fits (blue lines). Each dot summarizes a 20-hour timeseries experiment (see Figure 2).

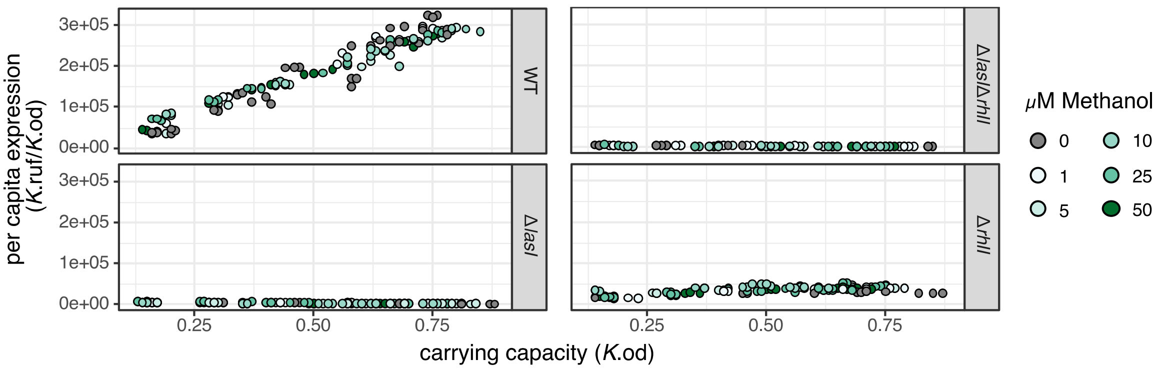

Supplemental Figure 2. Methanol does not impact expression or growth. In the case of supplementing signal, the AHL signaling molecules used in this study are suspended in methanol. Therefore, we first tested if different concentrations of methanol impacted our experiments and found that overall, methanol did not significantly impact (ANOVA, df = 500, p = 0.627) or expression (ANOVA, df = 500, p = 0.133).

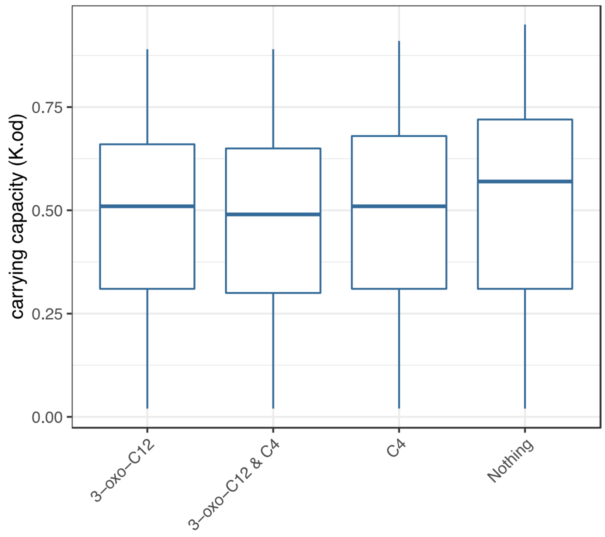

Supplemental Figure 3. Exogenous signal addition does not significantly impact carrying capacity in the WT (ANOVA, $F(3,469) = 1.665$, $p = 0.174$).

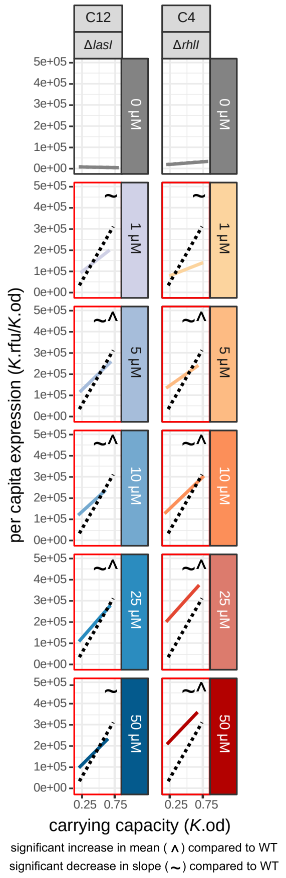

Supplemental Figure 4. Cognate signal supplementation compared to the WT expression. In most comparisons, mean expression is significantly higher than WT (Dunnett's Test, df = (10,353), $p_{lasI+50uM3\text{-}oxo\text{-}C12} = 0.543$, $p_{lasI+1uM3\text{-}oxo\text{-}C12} = 1$, $p_{rhlI+1uMC4} = 1$, otherwise $p < 0.001$), but slope is significantly lower compared to the WT (Dunnett's Test, df = (10,353), $p < 0.001$ for all comparisons).
